# *In vivo* dual-mode full-field optical coherence tomography for differentiation of types of melanocytic nevi

**DOI:** 10.1117/1.JBO.26.2.020501

**Published:** 2021-02-23

**Authors:** Ming-Rung Tsai, Tuan-Shu Ho, Yu-Hung Wu, Chih-Wei Lu

**Affiliations:** a Apollo Medical Optics, Ltd., Taipei, Taiwan; b Mackay Memorial Hospital, Department of Dermatology, Taipei, Taiwan; c Mackay Medical College, Department of Medicine, New Taipei City, Taiwan

**Keywords:** melanocytic nevus, optical coherence microscopy, *in vivo* imaging, cellular resolution

## Abstract

**Significance:** Melanocytic nevi represent the most common dermal melanocytic lesions in humans. Nevus is typically diagnosed clinically with the naked eye or with dermoscopy. However, it is essential to identify the type of nevus by invasive biopsy for histopathological examination. The use of noninvasive imaging tools can be used to evaluate the types of nevi to reduce unnecessary excisions of benign entities.

**Aim:** To evaluate the feasibility of using *en face* and cross-sectional full-field optical coherence tomography (FF-OCT) in differentiation of melanocytic nevi that can facilitate the reduction of unnecessary excisions of benign entities.

**Approach:** Dual-mode Mirau-type FF-OCT for cross-sectional imaging (B-scan) and *en face* imaging were used to distinguish the types of nevi.

**Results:** Although the B-scan reveals the distribution of melanosomes, users can set a specific depth of the *en face* image to explore the morphology of surrounding skin cells instantly. According to the locations of nevus nests, the different types of nevi, including junction nevus and compound nevus, can be identified using this dual-mode FF-OCT system.

**Conclusions:** Combining B-scan and *en face* imaging *in vivo* FF-OCT enables the examination and navigation of skin tissues in real time and in three dimensions.

## 1 Introduction

Melanocytic nevi are common skin lesions in humans. Nevus can be classified as either acquired or congenital according to the onset. They can be further subdivided into junctional, compound, and intradermal nevus based on the histological location and distribution of the proliferating melanocytes in the epidermis and/or dermis.^1^ Several studies have reported that ∼25% to 33% of cutaneous melanomas arise from nevi.^2^^,^^3^ The distinctions between the different histological types of nevi and between nevus and melanoma were based on clinical history, gross morphology, and histopathological features.^4^^,^^5^ In clinical practice, nevus can be diagnosed with the naked eye.^6^ The use of dermoscopy by physicians can provide further assessment of clinically atypical melanocytic lesions compared to naked-eye examinations.^7^ The dermoscopy aids in determining the distribution of pigments and blood vessels in the epidermis and papillary dermis, enabling physicians to assess the degree of atypia. However, the gold standard for confirming the type of nevi or melanoma is based on invasive biopsy in histopathological examinations. Newly developed noninvasive imaging tools can provide alternative methods for optical biopsy and enhance the reduction of unnecessary excisions of benign entities.

Recently, several advanced imaging tools have become available for *in vivo* skin imaging including confocal microscopy^8^^–^^12^ and optical coherence tomography (OCT).^13^^–^^15^ Confocal microscopy provides unique functions for noninvasive, *in vivo* assessment of human skin structures using *en face* imaging with lateral resolution of 1  μm.^12^ However, confocal microscopy distinguishes between skin cell layers with difficulty owing to its low axial resolution of ∼4  μm.^12^ A lateral resolution of 5 to 10  μm is more typical in conventional OCT technology.^13^^–^^16^ It is challenging to reveal the structure in cellular level. High-resolution OCT can provide an excellent imaging technique for examining cellular characteristics and quantifying the properties of skin structures including full-field OCT (FF-OCT)^17^^–^^19^ and line-field confocal OCT (LC-OCT).^20^^,^^21^ Although FF-OCT is a good imaging tool for *en face* images, the frame rate of cross-sectional imaging (B-scan) is limited by the low brightness of light sources such as halogen lamps and light emitting diodes.^18^^,^^19^ LC-OCT is a recently introduced technique for B-scan using a line camera and a supercontinuum light source that provides high-brightness capability for OCT imaging. An *en face* imaging of LC-OCT could be achieved using a mirror galvanometer for lateral scanning.^20^ However, as compared to one-dimensional scanning, higher *en face* rates can be achieved by FF-OCT with two-dimensional (2D) parallel detection owing to the commercially available 2D image sensors with high pixel rates.^22^^,^^23^ In addition, an FF-OCT that utilizes a 2D camera can address speckle noise because of spatial compounding.^24^

In this study, we used a high-brightness light source from Ti:sapphire crystal fiber that provides broadband emission for FF-OCT imaging. For the light source, our FF-OCT revealed *en face* imaging with a lateral resolution of ∼1  μm, and it also increased the frame rate of the B-scan. We present the concept of dual-mode Mirau-type FF-OCT, indicating the B-scan and *en face* imaging for clinical applications using a simple switch and a flexible probe. As indicated, it is essential to identify the types of nevi for the clinical diagnosis of melanocytic lesions. Therefore, we utilized the dual-mode FF-OCT system to obtain images of normal skin and two types of nevi in the same volunteer for clinical evaluation. Our system can switch between B-scan and *en face* imaging modes by simply clicking a button that enables clinicians to efficiently explore the nevus. The B-scan images show the distribution of melanosomes and users can set specific depths of the *en face* images to explore the morphology of the surrounding skin cells instantly. According to the location of the nevus nest, nevus, such as junction nevus, and compound nevus can be identified. Combining B-scan and *en face* imaging, *in vivo* FF-OCT enables the examination and navigation of skin tissues in real time and in three dimensions.

## 2 Methods

In this system (ApolloVue S100, Apollo Medical Optics, Ltd., Taipei, Taiwan), we used a glass-clad Ti:sapphire crystal fiber to generate broadband amplified spontaneous emission as a light source.^25^^–^^27^ The Ti:sapphire crystal fiber was pumped by two 520-nm laser diodes. The center wavelength and bandwidth are 780 and 150 nm, respectively, yielding an OCT system with a high axial resolution of 1.3  μm.^28^ The power of the light source was ∼20  mW and the power on the patient was about 5 mW. For the safety of the system, the light source was test according to both IEC 60825-1 and IEC 62471. The system was Federal Drug Administration (FDA)-approved as a class II medical device. The bright continuous-wave emission of the crystal fiber source is significantly advantageous for clinical applications compared to pulsed light sources owing to the signal-to-noise ratio (SNR) of FF-OCT being proportional to the average power of the light source.^28^^,^^29^

**Fig. 1:**
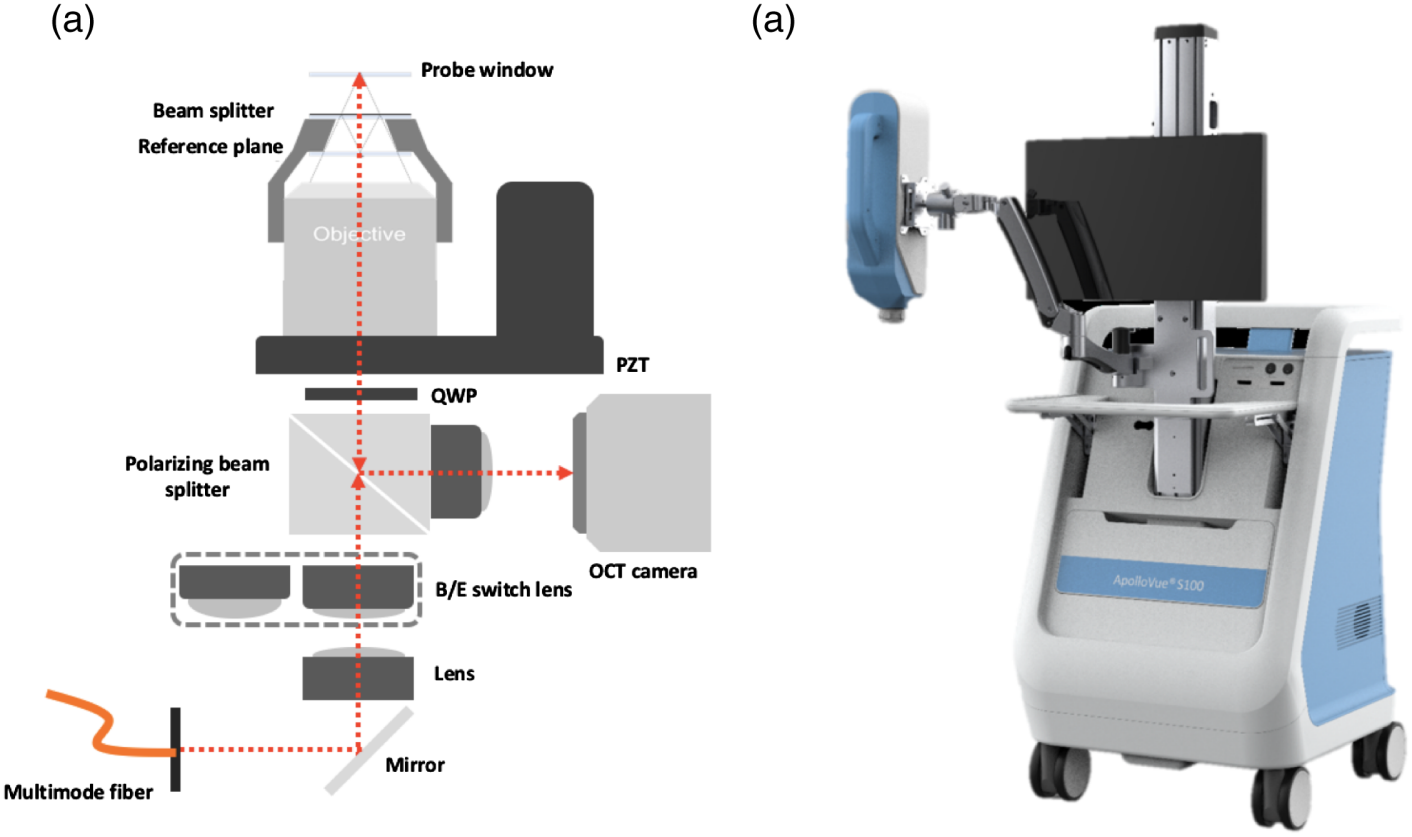
(a) Schematic of dual-mode Mirau-type FF-OCT system: PZT, piezoelectric stage and QWP, quarter wave plate. (b) Dual-mode Mirau-type FF-OCT system (ApolloVue S100, Apollo Medical Optics, Ltd., Taipei, Taiwan). The system was integrated with a flexible probe that can reach most parts of the skin.

A Mirau-type OCT configuration was adopted to minimize external disturbances such as environmental vibration and sample movement, as shown in Fig. 1(a). The broadband light was delivered into a multimode fiber and reflected by a polarizing cubic beam splitter. The Mirau objective was mounted on a piezoelectric (PZT) stage and the total travelling range of the PZT with open-loop control is 400  μm. The B-scan and *en face* images can be obtained by demodulating the interference signals acquired during PZT scans.^28^ The illumination module was integrated with a motorized slider to switch different lens modules to generate strip-field and wide-field illumination for B-scan mode and *en face* imaging mode, respectively.^30^ The Mirau objective was used to illuminate the subject’s skin and collect the back-scattered light from the sample and the reference arms. The back-scattered light beams from the sample and reference arms were combined after broadband polarizing cubic beam splitter and projected onto a 2D camera to generate the interferometric signals. A home-made 20× oil-immersion objective lens (NA: 0.80) was customized to correct the chromatic and spherical aberration introduced by the glass plates (reference plane and beamsplitter) and used to achieve a lateral resolution of 1  μm.^28^ To compensate for the chromatic dispersion of the subject, the Mirau objective was oil immersed. The field of views (FOV) of B-scan and *en face* images were $500\times 400\;{\mu m}^{2}$ (imaging depth) and $500\times 500\;\mu m^{2}$, respectively. The depth for imaging was controlled by the PZT stage. FF-OCT with a high-brightness light source, and a 2D camera can provide a frame rate of 0.43 fps in B-scan and 7 fps in *en face* imaging with cellular resolution. No frame averaging was performed. For clinical usability, the dual-mode FF-OCT system was integrated with a flexible probe that can reach most parts of the skin, as shown in Fig. 1(b). The probe was mounted on a swing arm that is flexible to rotate 360 deg in the sagittal plane and 180 deg in both the frontal and horizontal planes.

To demonstrate the capability for clinical applications using the *in vivo* dual-mode OCT system, we obtained images of a normal skin and two melanocytic nevi from the same volunteer (37-year-old, female) to observe the features of images. Nevus 1 and nevus 2 are on the right forearm and the right leg of the volunteer, respectively. The system also provided guiding images using an embedded video dermoscope. The use of guiding images can aid users to navigate observed subjects. Therefore, we used the guiding image to note the skin area of interest and switch to the FF-OCT mode to obtain the OCT images. From the features of images, the normal skin and nevus types can be identified.

## 3 Results and Discussion

A Mirau-type FF-OCT with a ${\mathrm{Ce}}^{3+}:\mathrm{YAG}$ crystal fiber as a light source for obtaining images of *in vivo* skin has been reported.^28^ The imaging depth using ${\mathrm{Ce}}^{3+}:\mathrm{YAG}$ crystal fiber is ∼100  μm owing to its wavelength of 560 nm. It is, therefore, difficult to show the structure of the dermis. In this study, we used Ti:sapphire crystal fiber as a light source. FF-OCT using Ti:sapphire crystal fiber with a wavelength of 780 nm can provide greater imaging depth.

**Fig. 2:**
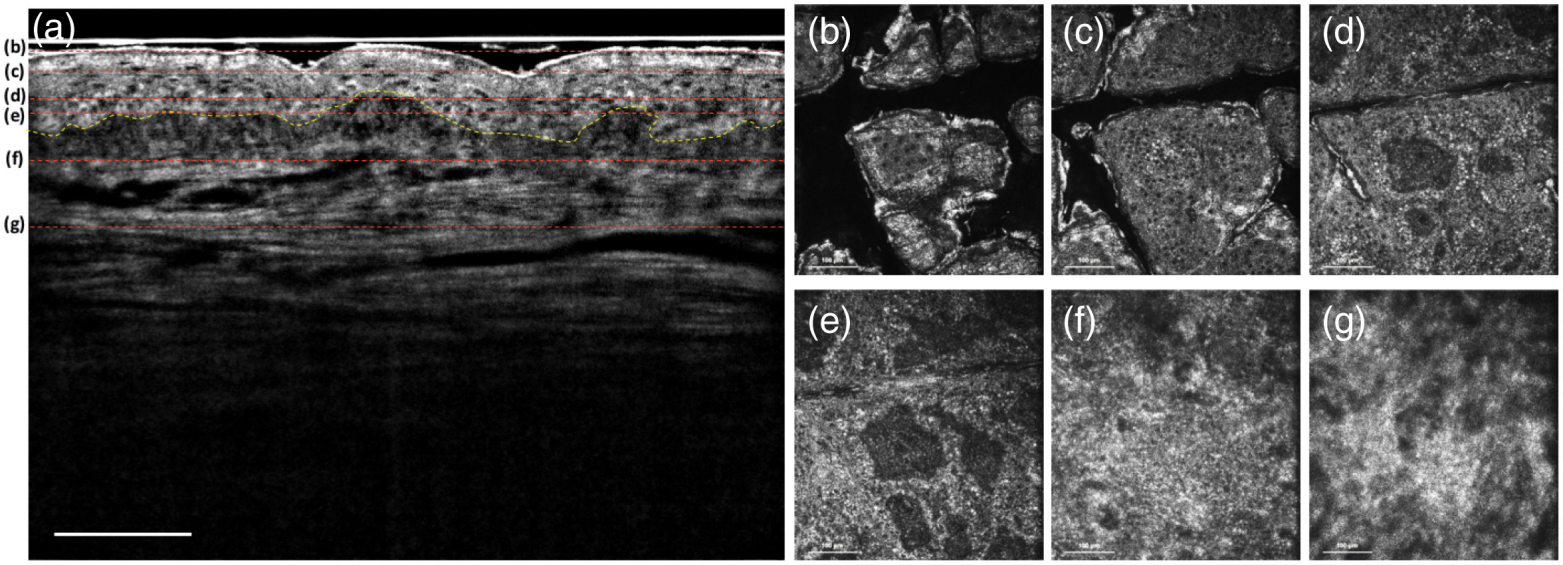
Normal skin: (a) *in vivo* FF-OCT B-scan image. The dashed yellow line presents the DEJ. (b)–(g) *In vivo* FF-OCT *en face* images at imaging depths of 30, 45, 65, 75, 110, and 160  μm, respectively. The red dashed lines in (a) show the different depths of the *en face* images from (b)–(g). From (b)–(e), the different layers of epidermis including the stratum corneum, stratum granulosum, stratum spinosum, and stratum basale can be obtained. (f) and (g) The papillary dermis and reticular dermis, respectively, which have different structures of collagen fibers. Scale bar: 100  μm.

Figure 2(a) shows the *in vivo* FF-OCT B-scan image of the normal skin on the forearm, and the imaging depth that can be achieved is over 250  μm. The B-scan image of human skin shows a vertical image including the epidermis, dermis, and dermal–epidermal junction (DEJ) with cellular resolution simultaneously. The dashed yellow line presents the DEJ, that is the region of skin between the epidermis and dermis.^31^ Our system yielded the *en face* images shown in Figs. 2(b)–2(g) at imaging depths of 30, 45, 65, 75, 110, and 160  μm, respectively. The different morphologies of keratinocytes are shown in Figs. 2(b)–2(e), including the stratum corneum, stratum granulosum, stratum spinosum, and stratum basale. The nuclei of keratinocytes were resolved in the epidermis and appeared as black holes in the images. Figures 2(f) and 2(g) show the papillary dermis and reticular dermis, respectively, which have different structures of collagen fibers.

**Fig. 3:**
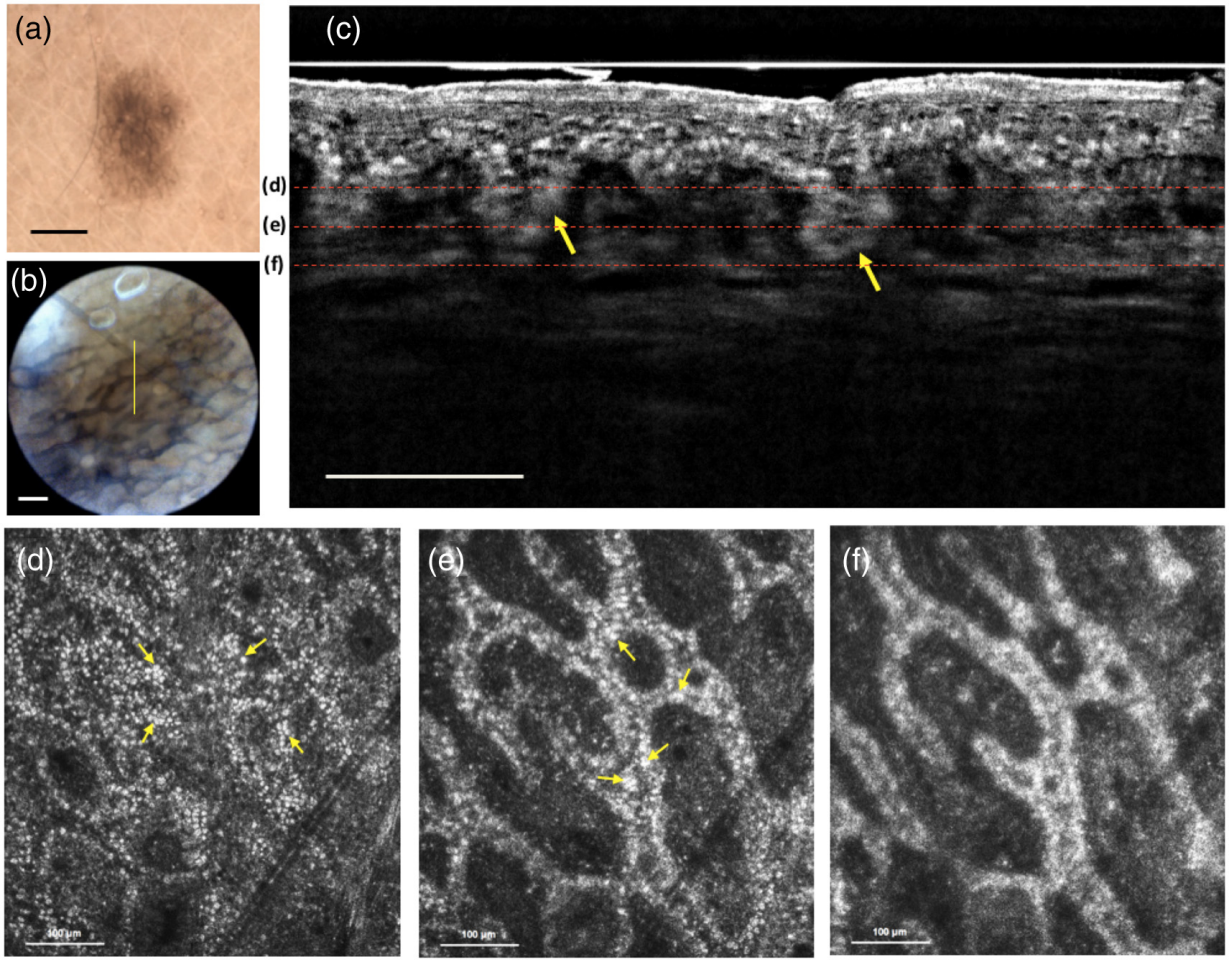
Junctional nevus: (a) dermoscopy image. Scale bar: 1 mm. (b) The guiding image. The yellow line represents the section region of the B-scan image. Scale bar: 200  μm. (c) *In vivo* FF-OCT B-scan image. The yellow arrows show the nevus nest in the DEJ. Scale bar: 100  μm. (d)–(f) *In vivo* FF-OCT *en face* images at imaging depths of 90, 110, and 130  μm, respectively. The red dashed lines in (c) show the different depths of the *en face* images from (d)–(f). The images show the elongation of rete ridges and increase hyper-reflective melanocytes. The yellow arrows in (d) and (e) indicate the hyper-reflective melanocytes.

Nevus 1 was on the right forearm of the volunteer. Dermoscopy evaluation of nevus 1 was performed with a digital dermoscope (DermLite Cam, DermLite) and an embedded video dermoscope (built-in S100 system), as shown in Figs. 3(a) and 3(b), respectively. The dual-mode FF-OCT images were obtained from the nevus shown in Figs. 3(c)–3(f). The yellow line in Fig. 3(b) represents the section region of the B-scan in Fig. 3(c). The B-scan image of nevus 1 shows the distribution of melanosomes near the DEJ, and the yellow arrows show the nevus nests in the DEJ. The depth of the nevus nest can be obtained near 100  μm. Therefore, we obtained *en face* images at imaging depths of 90, 110, and 130  μm, respectively, in Figs. 3(d)–3(f), and the images show the elongation of rete ridges and increased hyper-reflective melanocytes, which are features found in histology.^32^ By combining the features of B-scan and *en face* images, nevus 1 was considered as a junctional nevus.

**Fig. 4:**
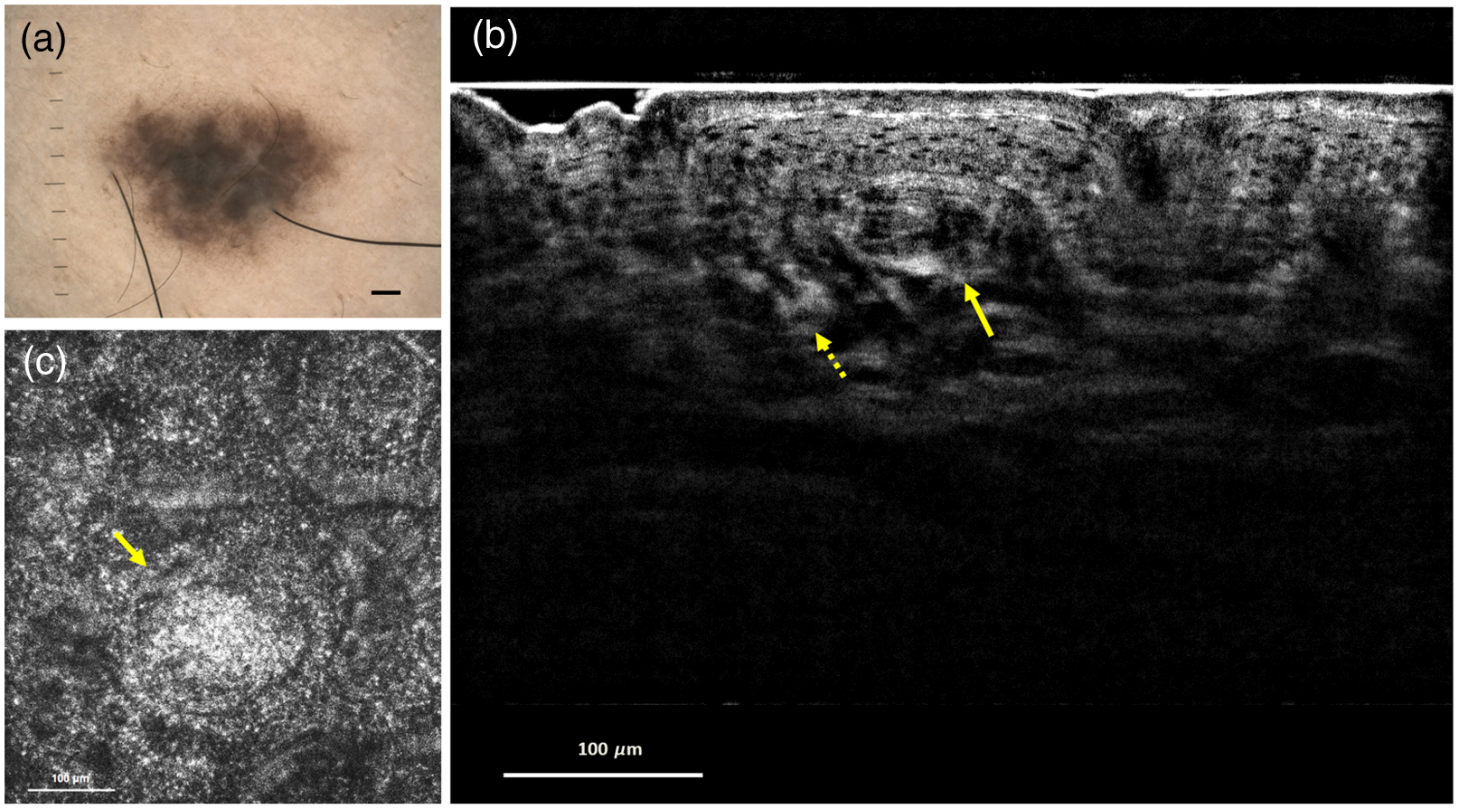
Compound nevus: (a) dermoscopy image. Scale bar: 1 mm. (b) *In vivo* FF-OCT B-scan image. The dashed and solid yellow arrows show the nevus nests in the basal cell layer and dermis, respectively. (c) *In vivo* FF-OCT E-scan image at an imaging depth of 120  μm. The yellow arrow shows the morphology of the nevus nest in the dermis.

Nevus 2 was on the right leg of the volunteer. Figure 4(a) shows the dermoscopy of nevus 2. Different dermoscopic findings, including shape, size, color, and distribution of pigmentation, were determined by comparing dermoscopic images of nevus 1 and nevus 2. An *in vivo* B-scan image is shown in Fig. 4(b). The dashed and solid yellow arrows show the nevus nests in the basal cell layer and dermis, respectively. The nevus nest in the dermis is at an imaging depth of 120  μm from the B-scan image of nevus 2. Therefore, we obtained the *en face* images of nevus 2 at this imaging depth and the *en face* image provides the morphology of the nevus nest, as shown in Fig. 4(c). B-scan image indicated the locations of nevus nests both in DEJ and dermis, which is a histological feature of compound nevus.^33^ Nevus 2 was considered to be a compound nevus.

FOV and imaging depth are both important for dermatology applications. The FOV of our FF-OCT is $500\times 500\;{\mu m}^{2}$ for *en face* images. Although many lesions are larger than this size, the entire lesion image could be acquired by means of image stitching under the frame rate of 10 fps for *en face* imaging. As for the depth, it is known that cancers such as melanoma and basal cell carcinoma both originate and spread from DEJ, which is usually within 100  μm from the surface.^34^ From Figs. 3(c) and 4(b), it is noted that the DEJ can be clearly revealed in our FF-OCT images. In addition, the nevus nest at the imaging depth of 120  μm can be also observed in Fig. 4(c). Therefore, the FOV and imaging depth of the FF-OCT system are adaptable for dermatology applications.

Comparing the light source with the wavelength of 560 nm,^28^ the wavelength of 780 nm is away from the absorption peaks of melanin and hemoglobin. Therefore, the SNR of the images using the 780-nm wavelength can be higher than that using the 560-nm wavelength. According to the similar measurement method,^28^ the SNR of this system in the epidermis was generally higher than 30 dB.

From the results, combing the B-scan and *en face* imaging is essential for efficiently showing the structures of nevi. The B-scan view is natural for clinicians, because it is similar to histopathological sections and a significant depth can be obtained for the *en face* imaging at a specific depth to evaluate the cell morphology. In addition, although common melanocytic nevi are predominantly benign, thus cosmetic removal of facial nevi is a frequent procedure for cosmetic surgeons.^35^^,^^36^ The nevus depth is an important parameter that influences the success of laser therapy.^37^ This FF-OCT imaging tool highlights the architecture and distribution of melanocytes and provides useful information for treatment. From the B-scan images of junction nevus and compound nevus in Figs. 3(c) and 4(b), the imaging depths of nevus nests can be obtained, increasing the success rate for nevus removal.

## 4 Conclusion

We present an FF-OCT system that provides imaging of human skin *in vivo* at cellular resolution in B-scan and *en face* imaging modes. Compared to the ${\mathrm{Ce}}^{3+}:\mathrm{YAG}$ crystal fiber generating a light source with a wavelength of 560 nm, this system used the Ti:sappire crystal fiber with a 780-nm wavelength light source, which provides a high imaging depth of human skin *in vivo*. The system adopted a flexible probe, with the possibility for the user to reach most parts of the skin. By clicking a button, the B-scan and *en face* imaging modes can be switched instantly. These features enable users to explore the skin efficiently.

To address the capability of clinical application, the dual-mode FF-OCT imaging was applied to normal skin and two different nevi for evaluation. Different cell layers in the epidermis, dermis, and clear DEJ can be obtained in the normal skin. From the imaging of the nevus, the B-scans showed the distribution of melanosomes, and users can set specific depths of the *en face* imaging to explore the morphology of surrounding skin cells instantly. According to the locations of nevus nests, the types of nevi, including junction nevus and compound nevus, can be identified using this dual-mode FF-OCT system. Combining B-scan and *en face* imaging, *in vivo* FF-OCT enables the examination and navigation of skin tissues in real time and in three dimensions. The dual-mode FF-OCT technique provides a powerful alternative tool and approach for optical biopsy and reduces unnecessary excisions of benign entities and further clinical applications of skin lesions.

## References

[1] Happle R., “What is a nevus? A proposed definition of a common medical term,” Dermatology 191(1), 1–5 (1995). 10.1159/000246468 8589475

[2] Bevona C. et al., “Cutaneous melanomas associated with nevi,” Arch. Dermatol. 139(12), 1620–1624 (2003). 10.1001/archderm.139.12.1620 14676081

[3] Lin W. M. et al., “Outcome of patients with de novo versus nevus-associated melanoma,” J. Am. Acad. Dermatol. 72(1), 54–58 (2015). JAADDB 0190-9622 10.1016/j.jaad.2014.09.028 25440436

[4] Damsky W. E., “Melanocytic nevi and melanoma: unraveling a complex relationship,” Oncogene 36(42), 5771–5792 (2017). ONCNES 0950-9232 10.1038/onc.2017.189 28604751 PMC5930388

[5] Elder D. E., “Precursors to melanoma and their mimics: nevi of special sites,” Mod. Pathol. 19, S4–S20 (2006). MODPEO 0893-3952 10.1038/modpathol.3800515 16446715

[6] Rigel D. et al., “The evolution of melanoma diagnosis: 25 years beyond the ABCDs,” CA Cancer J. Clin. 60(5), 301–316 (2010). CAMCAM 0007-9235 10.3322/caac.20074 20671054

[7] Holmes G. A. et al., “Using dermoscopy to identify melanoma and improve diagnostic discrimination,” Fed. Pract. 35(4), S39–S45 (2018). 30766399 PMC6375419

[8] Rajadhyaksha M. et al, “In vivo confocal scanning laser microscopy of human skin II: advances in instrumentation and comparison with histology,” J. Invest. Dermatol. 113(3), 293–303 (1999). JIDEAE 0022-202X 10.1046/j.1523-1747.1999.00690.x 10469324

[r9] Nehal K. S. et al., “Skin imaging with reflectance confocal microscopy,” Semin. Cutan. Med. Surg. 27(1), 37–43 (2008). 10.1016/j.sder.2008.01.006 18486023

[r10] Calzavara-Pinton P. et al., “Reflectance confocal microscopy for in vivo skin imaging,” Photochem. Photobiol. 84(6), 1421–1430 (2008). PHCBAP 0031-8655 10.1111/j.1751-1097.2008.00443.x 19067964

[r11] Ahlgrimm-Siess V. et al., “Confocal microscopy in skin cancer,” Curr. Dermatol. Rep. 7(2), 105–118 (2018). 10.1007/s13671-018-0218-9 29780659 PMC5956038

[12] Meschieri A. et al., “Reflectance confocal microscopy: a new tool in skin oncology,” Photonics Lasers Med. 2(4), 277–285 (2013). 10.1515/plm-2013-0027

[13] Alex A. et al., “Multispectral in vivo three-dimensional optical coherence tomography of human skin,” J. Biomed. Opt. 15(2), 026025 (2010). JBOPFO 1083-3668 10.1117/1.3400665 20459270

[r14] Sattler E. et al., “Optical coherence tomography in dermatology,” J. Biomed. Opt. 18(6), 061224 (2013). JBOPFO 1083-3668 10.1117/1.JBO.18.6.061224 23314617

[r15] Ulrich M. et al., “Dynamic optical coherence tomography in dermatology,” Dermatology 232(3), 298–311 (2016). DERMEI 0742-3217 10.1159/000444706 27104356

[16] Gamblicher T. et al., “Characterization of benign and malignant melanocytic skin lesions using optical coherence tomography in vivo,” J. Am. Acad. Dermatol. 57(4), 629–637 (2007). JAADDB 0190-9622 10.1016/j.jaad.2007.05.029 17610989

[17] Latrive A. et al., “In vivo and in situ cellular imaging full-field optical coherence tomography with a rigid endoscopic probe,” Biomed. Opt. Express 2(10), 2897–2904 (2011). BOEICL 2156-7085 10.1364/BOE.2.002897 22025991 PMC3191453

[r18] Dubois A. et al., “High-resolution full-field optical coherence tomography with a Linnik microscope,” Appl. Opt. 41(4), 805–812 (2002). APOPAI 0003-6935 10.1364/AO.41.000805 11993929

[19] Dubois A. et al., “Ultrahigh-resolution full-field optical coherence tomography,” Appl. Opt. 43(14), 2874–2883 (2004). APOPAI 0003-6935 10.1364/AO.43.002874 15143811

[20] Ogien J. et al., “Dual-mode line-field confocal optical coherence tomography for ultrahigh-resolution vertical and horizontal section imaging of human skin in vivo,” Biomed. Opt. Express 11(3), 1327–1335 (2020). BOEICL 2156-7085 10.1364/BOE.385303 32206413 PMC7075601

[21] Dubois A. et al., “Mirau-based line-field confocal optical coherence tomography,” Opt. Express 28(6), 7918–7927 (2020). OPEXFF 1094-4087 10.1364/OE.389637 32225427

[22] Auksorius E. et al., “In vivo imaging of the human cornea with high-speed and high-resolution Fourier-domain full-field optical coherence tomography,” Biomed. Opt. Express 11(5), 2849–2865 (2020). BOEICL 2156-7085 10.1364/BOE.393801 32499965 PMC7249809

[23] Ogien J. et al., “A compact high-speed full-field optical coherence microscope for high-resolution in vivo skin imaging,” J. Biophotonics 12(2), e201800208 (2019). 10.1002/jbio.201800208 30062826

[24] Avanaki M. R. et al., “Spatial compounding algorithm for speckle reduction of dynamic focus OCT images,” IEEE Photonics Technol. Lett. 25(15), 1439–1442 (2013). IPTLEL 1041-1135 10.1109/LPT.2013.2266660

[25] Hsu K. Y. et al., “Diode-laser-pumped glass-clad Ti:sapphire crystal fiber based broadband light source,” IEEE Photonics Technol. Lett. 24(10), 854–856 (2012). IPTLEL 1041-1135 10.1109/LPT.2012.2189202

[r26] Wang S. C. et al., “Broadband and high-brightness light source: glass-clad Ti:sapphire crystal fiber,” Opt. Lett. 40(23), 5594–5597 (2015). OPLEDP 0146-9592 10.1364/OL.40.005594 26625059

[27] Wang S. C. et al., “Laser-diode pumped glass-clad Ti:sapphire crystal fiber laser,” Opt. Lett. 41(14), 3217–3220 (2016). OPLEDP 0146-9592 10.1364/OL.41.003217 27420499

[28] Tsai C. C. et al., “Full-depth epidermis tomography using a Mirau-based full-field optical coherence tomography,” Biomed. Opt. Express 5(9), 3001–3010 (2014). BOEICL 2156-7085 10.1364/BOE.5.003001 25401013 PMC4230872

[29] Chen Y. T. et al., “En Face and cross-sectional corneal tomograms using sub-micron spatial resolution optical coherence tomography,” Sci. Rep. 8(1), 14349 (2018). SRCEC3 2045-2322 10.1038/s41598-018-32814-3 30254253 PMC6156507

[30] Ho T. S. et al., “In vivo Mirau-type optical coherence microscopy with symmetrical illumination,” Proc. SPIE 11228, 112280X (2020). PSISDG 0277-786X 10.1117/12.2544632

[31] Woodley D.T., “Importance of the dermal-epidermal junction and recent advances,” Dermatologica 174(1), 1–10 (1987). DERAAC 0011-9075 10.1159/000248972 3542613

[32] Gundalli S. et al., “Histopathological spectrum of benign melanocytic nevi—our experience in a tertiary care centre,” Our Dermatol. Online 7(1), 21–25 (2016). 10.7241/ourd.20161.5

[33] Nathan T. et al., “A practical approach to the diagnosis of melanocytic lesions,” Arch. Pathol. Lab. Med. 143(7), 789–810 (2019). 10.5858/arpa.2017-0547-RA 30059258

[34] Kurugol S. et al., “Pilot study of semiautomated localization of the dermal/epidermal junction in reflectance confocal microscopy images of skin,” J. Biomed. Opt. 16(3), 036005 (2011). JBOPFO 1083-3668 10.1117/1.3549740 21456869 PMC3077965

[35] Niamtu J., “Esthetic removal of head and neck nevi and lesions with 4.0-MHz radio-wave surgery: a 30-year experience,” J. Oral Maxillofac. 72(6), 1139–1150 (2014). 10.1016/j.joms.2013.10.015 24388180

[36] Sardana K. et al., “Optimal management of common acquired melanocytic nevi (moles): current perspectives,” Clin. Cosmet. Invest. Dermatol. 7, 89–103 (2014). 10.2147/CCID.S57782 PMC3965271 24672253

[37] Sardana K., “The science, reality, and ethics of treating common acquired melanocytic nevi (moles) with lasers,” J. Cutan. Aesthet. Surg. 6(1), 27–29 (2013). 10.4103/0974-2077.110093 23723601 PMC3663172

